# Effect of Multicomponent Exercise Program on Pain and Functional Mobility in Antitubercular Drug Therapy-Induced Peripheral Neuropathy in Pulmonary Tuberculosis Survivors

**DOI:** 10.7759/cureus.65431

**Published:** 2024-07-26

**Authors:** Tanisha Hiwalkar, Sandeep Shinde, Akshanda Dhumale

**Affiliations:** 1 Department of Musculoskeletal Sciences, Krishna College of Physiotherapy, Krishna Vishwa Vidyapeeth, Deemed to be University (KIMSDU), Karad, IND

**Keywords:** tuberculosis, peripheral neuropathy, pain, mobility limitation, exercise

## Abstract

Objectives: Exercise therapy is a pivotal component in the multidisciplinary approach to managing tuberculosis (TB)-related peripheral neuropathy (PN). A personalized exercise protocol maximizes therapeutic benefits while minimizing the risk of symptom exacerbation. This study aimed to determine the effect of multicomponent exercise programs on pain and functional mobility in antitubercular drug therapy-induced PN in pulmonary TB survivors.

Methods: In the approved experimental study, 110 participants with antitubercular study drug-induced PN were randomly assigned to two groups, and data were analyzed using IBM SPSS Statistics for Windows, Version 26 (Released 2019; IBM Corp., Armonk, New York, United States). The study’s purpose was to evaluate the efficacy of a multicomponent exercise program on PN symptoms.

Results: The study assessed a multicomponent exercise regimen's effectiveness in individuals with TB-related PN. The experiment group demonstrated noteworthy pain reduction (p < 0.0001), improvement in neurological symptoms (p < 0.0001), and better functional outcomes in the 12-minute walk test (p < 0.0001) and sit-and-reach test (p < 0.0001). Conversely, the control group exhibited less significant improvements. The low p-values indicate the intervention's effectiveness, emphasizing its impact on pain reduction, neurological symptoms, and functional abilities in comparing the experimental and control groups.

Conclusion: The study demonstrated the significant effectiveness of an eight-week multicomponent exercise program in individuals with pulmonary TB-related PN, showcasing notable improvements in pain reduction and functional mobility compared to a conventional single-component regimen in the control group.

## Introduction

Peripheral neuropathy (PN) is a complex disorder involving dysfunction in over 100 types of nerves, leading to various signs of nerve damage. It is a multidimensional illness characterized by disruptions in nerve function and structure, primarily triggered by insults to the nerve body or myelin sheath. These insults initiate physiological changes resulting in the loss of normal nerve function and impairments in the affected individual [1].

Tuberculosis (TB) is frequently identified as the reason for neuropathy in relation to an ongoing or prior TB infection. The link emphasizes the infection's systemic impact, with the immunological response and granuloma development leading to nerve damage [2]. Exercise therapy is a component of the care given to patients with PN, especially in the later phases of recovery [3]. Numerous studies have been conducted to ascertain the prevalence of neuropathy, especially in patients with drug-susceptible TB (DS-TB). The results of these studies produced various rates ranging from 0% to 10% [4]. The occurrence of isoniazid-induced PN has been researched in the medical literature, with one study conducted by Ellard in 1984 indicating a rate of 0.2% [5].

Anti-TB drug-induced neurotoxicity manifests with recognized symptoms, primarily tingling and numbness in the feet. These sensations spread proximally and may worsen with prolonged treatment, leading to myalgias, muscular weakness, and ataxia [6]. Slow acetylators, metabolizing isonicotinic acid hydrazide (INH) at a slower pace, possess an increased chance of PN due to cumulative INH exposure, with about two and a half times higher area under the INH serum curve. This toxicity is worsened by vitamin B6 deficiency. The proposed strategy to mitigate this toxicity suggests using 6 mg of pyridoxine (vitamin B6) daily. This preventive approach aims to counteract the potential neurotoxic effects of INH, emphasizing the importance of nutritional supplements in optimizing TB therapeutic outcomes [7 -13]. The immune system's response to TB may unintentionally target peripheral nerves, causing neuropathic symptoms [14]. Granulomas, immune cell clusters, can form in nerves due to TB, exerting pressure. Although rare, there have been reports of deposition disease in isolated cases of TB-related optic neuritis [15].

There have been concerns expressed about the potential synergistic impact of PN and TB on patients. A comprehensive healthcare approach is crucial in tackling the occurrence of PN in the context of TB [16-17]. Patients with TB face compromised immune systems, malnutrition, and systemic inflammation. Specific medications, such as isoniazid and ethambutol (EMB), are linked to neurotoxicity. Although they are necessary for treating primary infection, their side effects emphasize the balance required in TB treatment to achieve benefits without causing harm [18]. Individuals with TB may develop various neuropathies because of the illness or the course of therapy, notably tenosynovitis and carpal tunnel syndrome [15]. Antitubercular medication-induced neurotoxicity can occasionally lead to severe, long-lasting symptoms such as atrophy and muscle fasciculations, usually later in the illness [19].

Vitamin B1 (thiamine), B6 (pyridoxine), B9 (folate), and B12 (cobalamin) deficiencies can cause neuropathic symptoms ranging from tingling and numbness to more serious problems [20]. A complex health concern exists at the junction of PN, TB, and diabetic mellitus (DM). The consequences of PN can lead to functional impairment and an increased risk of complications, including foot ulcers and infections. Chronic alcohol intake impairs immunity, increasing a person's vulnerability to infections like *Mycobacterium tuberculosis*. It is related to nutritional inadequacies, reduced cough reflexes, and mucosal damage, all of which contribute to an environment permissive to TB infection and development [21].

The standard pharmacological regimen for TB treatment consists of a mix of drugs, INH, rifampin (RIF), pyrazinamide (PZA), and EMB. However, INH and EMB have both been associated with the development of neuropathy. Isoniazid is believed to lead to the depletion of vitamin B6 (pyridoxine), an important cofactor for nerve function. EMB is thought to impact the optic nerve, resulting in vision problems [22]. The possibility of neuropathy becomes significant in the setting of TB patients with HIV coinfection; because antiretroviral medication (ART) is frequently a critical component of treatment. Although the mechanisms by which nucleotide reverse transcriptase inhibitors (NRTIs) cause neuropathy are unknown, mitochondrial toxicity and oxidative stress are believed to play an integral part in damage to the nerves [23-24].

Exercise therapy is crucial in treating neuropathies, especially during the transition from acute to chronic phases. It involves building both strength and stamina through targeted workouts to counteract muscular weakness, enhance coordination, and mitigate secondary limitations linked to neuropathy. A personalized approach ensures the optimization of exercise benefits while minimizing the risk of exacerbating symptoms by tailoring treatments to individual needs and capabilities.

Pitfalls regarding the study are as follows: Limited number of studies have been performed focusing on the pain and functional mobility in patients with PN secondary to antitubercular drug therapy. There is a lack of standardized multicomponent exercise program for individuals suffering from pain and functional mobility in PN due to antitubercular drug therapy. More research may be needed to understand the underlying mechanisms of how multicomponent exercise program helps to reduce symptoms of PN in a specific population. The aim was to find out the effectiveness of multicomponent exercise program in comparison to the conventional exercise program on pain and functional mobility in antitubercular drug therapy-induced PN in TB survivors.

## Materials and methods

The performed study was a randomized controlled trial, conducted in Krishna College of Physiotherapy, Karad. The study was approved by the institutional ethical committee of Krishna Vishwa Vidyapeeth, Karad, India (Protocol Number 053/2023-2024). It involved 110 male and female participants. Participants’ permission to be in the experiment was obtained by signing the consent form. Randomization allocated individuals into control and experimental groups, with data analyzed using the IBM SPSS Statistics for Windows, Version 26 (Released 2019; IBM Corp., Armonk, New York, United States). The pretest assessment of all the parameters was done. The exercise protocol for the experimental group involved warm-up, aerobic exercises, stretching, sensory integration, functional exercise, and proprioceptive training. The exercise protocol for the control group was more conventional including warm-up, deep breathing exercises, and free exercise for major joints. Individuals with a normal body mass index (BMI) of 18.5-24.9 who had received antitubercular medication therapy met the inclusion criteria. The standard duration for antitubercular drug therapy is 9 months. The age group of 25 to 45 was targeted to minimize age-related influences on PN, avoiding potential confounding factors beyond this range. The study duration was eight weeks, and a 45-minute session was conducted four times a week.

To ensure population homogeneity, pregnant women, individuals with underlying comorbidities, and those with pre-existing neuropathy were excluded from the study. This selective approach aimed to create a focused cohort for investigating the effectiveness of a multicomponent exercise regimen exclusively in patients who had undergone antitubercular medication therapy and exhibited symptoms of PN. The impact was assessed using criteria such as pain rating visual analog scale (VAS), modified total neuropathy score (mTNS), sit-and-reach test, and 12-minute walk test, which comprehensively evaluated pain perception, neuropathic symptoms, flexibility, and functional ability.

Manual and SPSS Version 26.0 statistical software were both employed for statistical analysis. The paired t-test was utilized to analyze the pre- and post-intervention data within the group, while an unpaired t-test was used for analyzing pre- and post-intervention data between the groups.

Outcome measures

Visual Analog Pain Rating Scale

Many scales are routinely used to assess pain, with the numeric rating scale (NRS), VAS, and verbal rating scale (VRS) being particularly popular in clinical practice. Empirical research has demonstrated the reliability and validity of various pain-rating methods in evaluating pain intensity [25].

mTNS

mTNS is a clinical diagnostic instrument for determining the severity of PN. The inter- and intrarater reliability is 0.966 and 0.986, respectively [26].

Sit-and-Reach Test

The sit-and-reach test is widely utilized to examine the flexibility of the lower back and hamstrings. The reliability of this test has intraclass correlation coefficient (ICC) of 0.91-0.93 [27]. 

12-Minute Walk Test

The 12-minute walk test is a prominent and standardized method for assessing aerobic capacity, cardiovascular fitness, and endurance (Tables 1-2) [28].

**Table 1:** Study group exercise protocol

	Warm-up	Aerobic exercise (8-10 min)	Range-of-motion exercise (all major joints)	Functional exercise and proprioceptive training	Sensory integration	Strength training (all major joints)	Stretching (major upper and lower limb muscles)
Week 1	5 min	Slow walking	5 x 2	Sit to stand, step back and forth, sidewalk	Exercises for stereognosis, temperature differentiation, grip training exercises (eyes open)	5 x 2 Using elastic band	2 x 10s hold
Week 2	5 min	Brisk walking	7 x 3	Half squat with wall support, half lunge, half side lunge	Exercises for stereognosis, temperature differentiation, grip training exercises (eyes closed)	5 x 3 Using elastic band	3 x 10s hold
Week 3	10 min	Spot jogging	7 x 3	Half squat, full lunge, full side lunge	Brushing protocol (10 x 2)	7 x 3 Using dumbbell (0.5kg)	2 x 15s hold
Week 4	10 min	Running	10 x 3	Full squat, full lunge and hold, side lunge and hold	Brushing protocol (10 x 3)	10 x 3 Using dumbbell (0.5/1 kg)	3 x 20s hold

**Table 2:** Control group exercise protocol

	Warm-up	Range-of-motion exercise (all major joints)	Strength training (all major joints)	Stretching (major upper and lower limb muscles)
Week 1	5 min	5 x 2	5 x 2 Using elastic band	2 x 10s hold
Week 2	5 min	7 x 3	5 x 3 Using elastic band	3 x 10s hold
Week 3	10 min	7 x 3	7 x 3 Using dumbbell (0.5kg)	2 x 15s hold
Week 4	10 min	10 x 3	10 x 3 Using dumbbell (0.5/1 kg)	3 x 20s hold

## Results

This study sought to evaluate the efficacy of a multicomponent exercise regimen in individuals with a history of TB who experienced symptoms of PN following anti-tubercular drug therapy. A total of 110 individuals aligning the inclusion criteria were randomly split into a study group (n = 55) and a control group (n = 55). The study group underwent the multicomponent exercise regimen, while the control group followed a standard exercise protocol. The main outcome measure employed was the mTNS. Statistical analysis revealed an exceptionally significant difference (p < 0.0001) between the two groups, indicating a positive impact of the multicomponent exercise regimen on PN.

We designed an eight-week exercise program for the study group, covering various aspects of physical fitness. It included warm-up, aerobic exercises, isometric exercises, free exercises, stretching, sensory integration, functional exercises, proprioceptive training, and strength training. The initial two weeks focus on warm-up, aerobic exercise, isometric exercises, free exercises, and stretching. Intensity increases with brisk walking, longer isometric exercises, and additional sets by the fourth week. Specific movements and brushing protocols are introduced. Participants advance to spot jogging and more challenging exercises in the sixth week, including sensory integration. By the eighth week, they progress to running, intense isometric and free exercises, and challenging sensory integration with closed eyes. Strength training intensifies with weights in exercises like full squats and lunges.

Table 3 displays a varied age distribution in the study population, with the majority falling within younger age brackets. 

**Table 3:** Demographic variables

Parameter	Frequency	Percentage
Age group		
25-30	28	25.45%
31-35	20	18.18%
36-40	15	13.6%
41-45	47	42.72%
Gender		
Male	73	66.33%
Female	27	33.66%
Population		
Rural	68	61.8%
Urban	42	38.1%

Table 4 displays the VAS score. Our objective was to measure pain levels using the VAS before and after a specific course of action. The VAS is a subjective metric that allows people to indicate the severity of their pain on a scale. The study group exhibited notable improvements in pain scores. In contrast, the control group had a mean pain score at rest of 3.72 before the intervention, which slightly decreased to 3.63 after a specific interval. The study group showed a significant reduction in pain levels at rest and during activity following the intervention (p < 0.0001). In comparison, people who were in the control group failed to notice a significant drop in pain levels at rest (p = 0.0004), and the difference throughout the activity was not statistically significant (p = 0.2773). These data indicate that the intervention in the study group was highly effective in pain reduction, as indicated by the extremely significant p-values.

**Table 4:** Comparison of the VAS score VAS: Visual analog scale

Study group			
Pre-test			
	Visual analog scale (on rest)	Visual analog scale (on activity)	p-value
Mean	3.45 ± 1.22	7.14 ± 1.53	p < 0.0001
Post-test			
Mean	1.70 ± 1.14	4.10 ± 1.13	
Control group			
Pre-test			p = 0.004
Mean	3.72 ± 1.48	6.27 ± 2.10	
Post-test			
Mean	3.63 ± 1.04	6.05 ± 1.29	

Table 5 compares the mTNS in the study group before and after the multicomponent exercise program. Prior to the intervention, the overall neurological symptom score was 9.83. Consequently, the overall neurological symptom score significantly decreased to 6.67 post-intervention. P-values for sensory symptoms, motor symptoms, superficial sensitivity, vibratory sensitivity, and strength were highly significant (<0.0001, <0.001, <0.0001, <0.0001, 0.0004, respectively), while the p-value for reflexes was 0.1592, considered nonsignificant. Further developments in this aspect possibly provide insightful information on potential improvements. The p-value < 0.0001 for the total neuropathy score in the study group indicates a significant decrease in neurological symptoms after the intervention in the study group.

**Table 5:** Comparison of the modified total neuropathy score

	Study group		
	Pre-test	Post-test	p-value
Sensory symptoms	2.69 ± 0.92	1.56 ± 1.04	<0.0001
Motor symptoms	2.12 ± 0.66	1.25 ± 0.77	<0.001
Superficial sensitivity	1.65 ± 0.67	1.21 ± 0.76	<0.0001
Vibratory sensitivity	1.01 ± 0.62	0.67 ± 0.63	<0.0001
Strength	1.89 ± 0.85	1.58 ± 0.85	<0.0004
Reflexes	0.45 ± 0.50	0.38 ± 0.49	0.15192
Total	9.83 ± 3.07	6.67 ± 1.96	<0.0001
	Control group		
Sensory symptoms	2.25 ± 0.98	2.10 ± 0.99	0.138
Motor symptoms	2.09 ± 0.79	1.90 ± 0.72	0.0064
Superficial sensitivity	1.69 ± 0.79	1.6 ± 0.73	0.2285
Vibratory sensitivity	1.05 ± 0.55	0.94 ± 0.59	0.1095
Strength	1.92 ± 0.93	1.78 ± 0.87	0.0312
Reflexes	0.52 ± 0.50	0.47 ± 0.50	0.0832
Total	9.54 ± 3.20	8.81 ± 2.52	0.0007

In the pre-test phase, the control group had an average of 9.54 for the total neuropathy score. Post-test scores showed slight changes after the intervention. The overall neurological symptom score slightly declined to 8.81. In the control group, sensory symptoms had a nonsignificant p-value of 0.138. Motor symptoms showed a marginally significant decrease (p = 0.0064): superficial (p = 0.2285, insignificant) and vibratory sensitivity (p = 0.1095, not significant). The strength parameter demonstrated a statistically significant drop (p = 0.0312) and reflexes (p = 0.0832, not significant). These results indicate a mixed response to the control group's intervention. The control group has a notable p-value of p = 0.0007 for the total score, although not as much as the study group (p < 0.0001). The unpaired t-test comparing the total neuropathy score between the study and control groups revealed a highly significant difference (p < 0.0001).

Table 6 depicts the results of the 12-minute walk test. Within the study group, paired t-tests showed a highly significant increase (p < 0.0001). In contrast, the control group yielded a p-value of 0.3836 (insignificant). Additionally, an unpaired t-test demonstrated a notable distinction between the study and the control groups (p = 0.0005). 

**Table 6:** Comparison of the 12-minute walk test

Study group		p-value
Pre-test	697.89 ± 30.60 m	<0.0001
Post-test	709.94 ± 30.33 m	
Control group		0.3836
Pre-test	685.41 ± 35.98 m	
Post-test	686.34 ± 37.93 m	

Table 7 presents the sit-and-reach test results. For the study group, p < 0.0001 indicates improved flexibility. In contrast, for the control group, p = 0.7387 is insignificant. The unpaired t-test showed no significant difference, yielding a p-value of 0.2184. This implies that the observed variations in flexibility between the two groups are not statistically significant.

**Table 7:** Comparison of the sit-and-reach test

Study group		p-value
Pre-test	6.18 ± 2.09 cm	<0.0001
Post-test	7.20 ± 2.02 cm	
Control group		
Pre-test	6.68 ± 2.22 cm	0.7387
Post-test	6.7 ± 2.24 cm	

## Discussion

High-dose INH has been identified as a potential contributor to an increased incidence of PN compared to conventional doses, as noted by Steichen [29]. The rapid incorporation of linezolid (LZD) into standard TB treatment can cause side effects such as PN as reflected by a study by Agyeman [30]. Streckmann et al. in their study involved twice-weekly sessions comprising aerobic, sensorimotor, and strength training components whose results did not indicate a significant difference in pain [31]. Our study yields strong proof of a positive impact on pain management in individuals with PN. In Kluding et al.'s study, a 10-week aerobic and strengthening exercise program resulted in a significant decrease in discomfort, neuropathic symptoms, and an increase in intra-epidermal nerve fiber branching [32]. Our study adopted a similar approach and showed a substantial reduction in neuropathic symptoms within the study group (p < 0.0001).

The mention of Naimat-Ullah et al. and Akbari et al. provide evidence for the idea that incorporating strengthening and balance training alongside aerobic exercises in diabetes-related PN is beneficial [32,33]. Our study integrates a combination of strength training and isometrics over an eight-week period, along with 10-15 minutes of aerobic exercises. It targets muscle function, stability, and cardiovascular health, addressing diverse aspects of physical well-being. According to Kanase et al., engaging in sensory integration exercises facilitates neural circuitry and promotes improved awareness of position sense, particularly in cases where kinesthesia may be disrupted. Furthermore, our study has incorporated sensory integration techniques with exercises aimed at enhancing stereognosis, temperature differentiation, and grip [34]. It seeks to offer a comprehensive strategy for addressing the challenges associated with neuropathy. In the study by Camarata et al., they integrated a sensory stimulation protocol involving brush stimuli and weighted vests. In our study as well, we have included a brushing protocol to enhance sensory integration [35]. A study by Holmes et al. on diabetic PN concluded that by giving proprioceptive training, there was significant improvement in the balance of the patient [36]. Our study substantiated the efficacy of proprioceptive training. The results showcased not only the alleviation of pain but also improvements in the static and dynamic balance of the patient.

Aligned with Streckmann et al.’s finding the patient may not be able to perform aerobic activities for longer periods, our study took a comprehensive approach to address PN symptoms, emphasizing the combined benefits of various exercise modalities. Hernandez-Secorun et al. showed the combination of aerobic exercise and manual therapy in the treatment of diabetic PN [37]. Our study incorporated resistance training using elastic bands, weight cuffs, and dumbbells for major joints along with aerobic exercises. This approach proved effective in alleviating neuropathy symptoms and contributed to an overall improvement in body strength. Nadi et al. demonstrated the importance of exercise as a cost-effective intervention for improving the condition of diabetic neuropathy patients. Their study highlighted exercise's profound and prolonged effects on glycemic control and inflammation. The authors emphasized the need to tailor exercise prescriptions, considering factors like wounds, leg numbness, sweating, and infection. The study suggested that exercise prescription for neuropathy (EPN) exercises, focusing on the lower limbs, were highly suitable for diabetic neuropathy patients [38]. Our study, conducted in conjunction with this research, expands the understanding of exercise interventions for individuals experiencing PN due to anti-tubercular drug therapy.

Gholami et al. stated that aerobic exercise training improved glucose control and nerve conduction velocity in sensory sural nerve. It demonstrated the possible advantages of sustained aerobic exercise to prevent diabetic PN [39]. The absence of comparable data in existing literature for people who experience PN due to anti-tubercular drugs motivated our comprehensive study. Tailoring our approach, we incorporated a mix of exercises, including both aerobic and resistance components. Addressing drug-induced PN issues, our research aims to provide insights into the potential efficacy of diverse exercise modalities in improving neuromuscular motor-sensory parameters, highlighting the importance of individualized exercise routines in treating various neuropathic etiologies.

As stated by Gherwara et al., TB disease and its treatment are associated with the reduced functional exercise capacity and decreased range of motion of major joints [40]. According to our research, TB survivors have substantial muscle weakness. In order to offset physical weakness and improve coordination, the routine entailed performing focused workouts to increase the strength of the muscles. In a study by Jagtap et al., it is noted that musculoskeletal impairments like pain, stiffness, and impairment in joint range of motion, are present post chikungunya viral infection [41]. In our study, we address impairments resulting from TB infection in this study, including functional capacity, proprioception, and muscle strength. Both studies contribute valuable insights into the multifaceted nature of musculoskeletal impairments following a viral infection. By synthesizing findings and exploring their implications for clinical practice and public health, researchers can advance our understanding and management of this significant health challenge.

This study has limitations, such as a relatively small sample size and a limited geographic scope, which could affect the generalized results. The study can be of longer duration so monthly follow-ups can be taken. Subsequent research should consider these factors when interpreting and applying the findings. There is potential for further academic exploration of this study, and opportunities exist for expanding the population under investigation.

## Conclusions

This study determined the effectiveness of a multicomponent exercise program in people with a history of pulmonary TB who were exhibiting post-drug therapy symptoms of PN. The results showed significant effectiveness of the multicomponent exercise program compared to the conventional single-component exercise regimen provided to the control group. Over eight weeks, participants in the multicomponent exercise group showed a substantial reduction in neuropathy symptoms, particularly experiencing lower pain levels and improved functional mobility. These findings imply that our carefully designed and progressively structured exercise program holds promise as an effective intervention for alleviating PN symptoms in individuals with a history of TB.
